# Two Postpartum Blood Collection Devices: The Brass-V Drape and MaternaWell Tray—As Experienced by Birth Attendants and Birthing Women—A Questionnaire-Based Randomised Study

**DOI:** 10.1155/2024/6605833

**Published:** 2024-08-14

**Authors:** Jade Esau, Timothy Morris, Chris Muller, Christine Els, Liesl de Waard

**Affiliations:** ^1^ Department of Obstetrics and Gynecology Faculty of Medicine and Health Sciences Stellenbosch University, Cape Town, South Africa; ^2^ Faculty of Medicine and Health Sciences Stellenbosch University, Cape Town, South Africa; ^3^ Department of Statistics and Actuarial Science Stellenbosch University, Stellenbosch, South Africa; ^4^ Department of Obstetrics and Gynecology Khayelitsha Hospital and Stellenbosch University, Cape Town, South Africa

## Abstract

**Background:**

Postpartum haemorrhage is the leading cause of preventable maternal mortality worldwide. Early identification and prompt management of postpartum haemorrhage improve outcomes. Objective assessment of postpartum blood loss is an important step in identifying postpartum haemorrhage. The Brass-V drape and MaternaWell tray have been designed for routine measurement of postpartum blood loss. The perceived utility and acceptability of these devices to the parturients and birth attendants still begged exploring.

**Objective:**

To assess the perceived usefulness and ease of use of a Brass-V drape versus a MaternaWell tray for the collection of postpartum blood loss.

**Methods:**

We conducted a prospective parallel randomised trial, employing a questionnaire to assess the experiences of birth attendants and birthing women who used these devices. The study was conducted at site B midwife obstetric unit in Khayelitsha Cape Town. Pregnant women presenting in early labour were approached for voluntary participation. After informed consent was obtained, participants were randomly assigned to the Brass-V drape or the MaternaWell tray, which the birth attendant placed after the birth of the baby.

**Results:**

There were 63 participants, of which 33 were assigned to the MaternaWell tray and 30 to the Brass-V drape. Birth attendants indicated a desire to use the MaternaWell tray (30 (90%)) or Brass-V drape (26 (87%)) in future deliveries. The parturients were also in favour of the future use of MaternaWell tray (33 (100%)) and Brass-V drape (28 (93%)). Ease of measurement favoured the Brass V-drape, and ease of placement favoured the MaternaWell tray. Five (8%) participants experienced postpartum haemorrhage, two with the MaternaWell tray and three with the Brass-V drape. One parturient required hospital transfer.

**Conclusion:**

The responses of the birth attendants and parturients were positive. The MaternaWell tray has the benefit of reuse and lower cost and is an acceptable alternative to the Brass-V drape. Both devices aid in the early recognition of postpartum haemorrhage.

## 1. Introduction

Postpartum haemorrhage (PPH), severe bleeding from the genital tract within 24 hours after childbirth, is the leading cause of maternal mortality worldwide [1]. Prevalence estimates vary from 1 to 10% in all births [2]. Obstetric haemorrhage (OH) accounts for 9.3% of maternal deaths in high-income countries and 45.7% in countries with low to middle income (LMIC) [3, 4]. In the triennial Saving Mothers report by the National Committee on Confidential Enquiries into Maternal Deaths (NCCEMD) in South Africa 2020–2022, obstetric haemorrhage was the second leading cause of maternal deaths in South Africa [5]. There were 599 deaths ascribed to OH with a maternal mortality ratio of 19.8/100 000 live births [5].

The World Health Organization defines PPH as a blood loss of ≥500 ml from the genital tract within 24 hours of vaginal birth [1]. Of the maternal deaths in the South African Saving Mothers report 2020–2022, OH was the second leading cause of maternal deaths in South Africa [5]. When PPH occurs, early identification of bleeding and prompt management with evidence-based interventions can avert most PPH-related severe morbidities and deaths [1, 6–8]. The recent landmark E-MOTIVE (early detection, massage of the uterus, oxytocic drug, tranexamic acid, IV fluid examination, and escalation) trial (a large international randomised trial of early detection and treatment of postpartum haemorrhage) showed that the use of a calibrated drape for detection of PPH and a bundling of simultaneous treatments resulted in a substantially lower risk of severe postpartum haemorrhage, laparotomy for bleeding, and death from bleeding than usual care in LMIC [8].

Different techniques have been used to quantify vaginal postpartum blood loss. There is insufficient evidence to support one method as superior to another when estimating blood loss after vaginal birth [9]. Although visual estimation is the most commonly practised measurement of blood loss, it has been proven to underestimate blood loss when there are large volumes and overestimate when there are smaller volumes [10, 11].

The Brass-V drape™ has shown great promise for use in LMIC, and its accuracy is proven with a 29–33% higher blood loss measurement compared to visual estimation [8, 12]. The blood collection drape consists of a funnelled and calibrated collecting pouch attached to a plastic sheet which is placed under the woman's buttocks immediately after birth [12]. Two belts attached to the upper end of the drape are tied around the woman's abdomen, and the sheet folds out to collect the placenta and blood loss. The MaternaWell tray™ (Maternova, Inc.) is a novel reusable tray in which blood loss is collected in a visible, calibrated 500 ml receiver for real-time monitoring and overflows to a second receiver alerts to PPH. The wedge is slipped under the parturient's buttocks so that her perineum is over the first calibrated receiver (similar to a “slipper” bedpan). A preliminary evaluation of acceptability and functionality conducted at the Frere Hospital's midwife obstetric unit, South Africa, concluded that it is acceptable to most birth attendants and participants and appears to function as intended [13].

To date, the perceived utility and acceptability of the MaternaWell tray, compared to the Brass-V drape, has not been assessed in a cohort of birth attendants and parturients.

## 2. Materials and Methods

We conducted a prospective parallel randomised trial from June to October 2022 at site B midwife obstetric unit (MOU) in Khayelitsha, Cape Town, South Africa. The aims were to assess the perceived utility and ease of use of a Brass-V drape [14] and an early prototype of the MaternaWell tray [13] for monitoring postpartum blood loss as assessed by the midwives and women giving birth [13].

Site B MOU is a midwife-led, public sector, birthing unit situated in Khayelitsha, Cape Town. Khayelitsha is one of the largest and fastest-growing townships with the majority of the population in informal settlements, representing mostly low-income households. The unit is staffed by birth attendants (trained nursing staff and midwives) and linked by telephone to the primary, secondary, and tertiary hospitals. They conducted 1070 births in 2022. The concept of the MOU is particularly suited to Africa and LMICs [15].

The study protocol was approved by the Health Research Ethics Committee of Stellenbosch University S21/10/191, and institutional permission was granted by the Western Cape Department of Health. The study was registered with the South African National Clinical Trials Registry (registration no. DOH-27-062022-9046). The South African Medical Research Council registration document with the Consort-2010 checklist is available as supplementary documents.

This was a pilot study with an aimed minimal sample size of 60 participants (30 in each arm). Pregnant women older than 18 years presenting with low-risk pregnancies at a gestational age of 36 weeks and more, planning to have a vaginal birth, were recruited to participate in the study. Convenience sampling was used. All potential participants were approached when they presented in early labour by the investigating team (the primary investigator and a research assistant). The investigating team recruited participants on days that they were available at the study site. The investigating team trained the birth attendants in the use of both devices, prior to the commencement of the study.

Voluntary, written, informed consent was obtained prior to enrolment in the study. Once the baby was born, participants were randomly assigned to either a Brass-V drape or a MaternaWell tray. Randomisation was performed electronically by allocating a device in a random sequence. The allocations were placed in opaque envelopes by the secretary. The investigating team and the birth attendants were blinded to the randomisation sequence. Allocation took place by drawing the next (consecutively numbered) sealed opaque envelope.

The birth attendants placed the allocated device underneath the parturient after the birth of the baby. The placenta was delivered onto the blood collection device and then removed. The midwife visually estimated the blood spillage on the draw sheet and measured the blood volume in the collection device. The blood collection device was removed once the bleeding had stopped or when 30 minutes had passed after placement. Along with the MaternaWell tray, a standard measuring jug was used to measure the blood. With the Brass-V drape, the measurement was read directly on the calibrations in accordance with the instructions of the drape and the device [16]. The midwife and parturient completed the questionnaire subsequent to the completion of the birth and postpartum blood collection measurements. Both received light refreshments as a gratuity for participating.

Descriptive and birth outcome measures were collected from the clinical records. The anonymised questionnaires and clinical data were entered into a password-protected Excel spreadsheet and analysed by SAS software, version 9.4 of the SAS System for Windows (copyright© 2024; SAS Institute Inc). Continuous variables were expressed as median values with interquartile ranges (IQRs) or mean with standard deviation (SD). Categorical variables were expressed as proportions (percent). Significance was defined as a *P* value of <0.05.

The primary outcome was the overall satisfaction (as rated on a questionnaire) of the birth attendants and parturients with the device (“Would you choose to use the device for future births?”). Clinical and descriptive data were collected as secondary outcomes.

## 3. Results

Sixty-three women consented and participated in the study. After randomisation, 30 were allocated the Brass-V drape (further referred to as “drape”) and 33 the MaternaWell tray (further referred to as “tray”) as blood loss monitoring devices. The median age (years) of the participants was 28 (IQR: 23–33) 28 (IQR: 25–33) in the drape arm and 27 (IQR: 23–31) in the tray arm. Most participants were multiparous, with a median gestational age in both groups of 39 (IQR 39-40) weeks. There were no differences in the parturient demographics. Further details are described in Table 1.

All participants had a vaginal birth of a singleton live baby. The mean duration of the second stage of labour was 47 minutes (SD: 73 min). The median birth weight was 3200 g (range: 2300–4000 g). There were no significant differences in the birth outcomes in the two groups, as demonstrated in Table 2.

**Figure 1:**
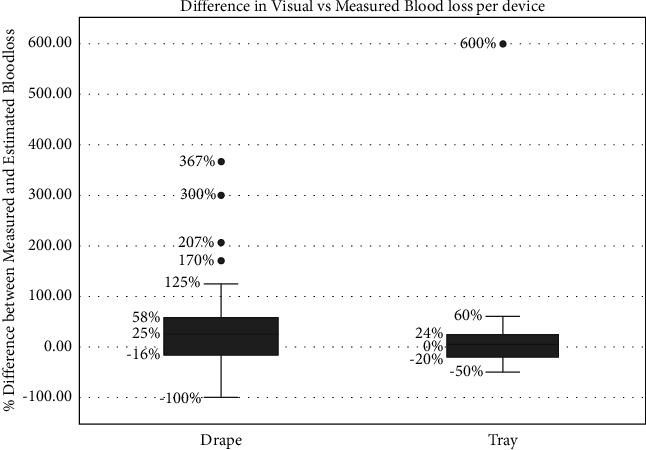
Percentage differences between measured and estimated blood loss.

The mean blood loss as visually estimated by the birth attendants was 212 ml (SD: 134) for all cases; that is, 203 ml in the drape arm and 220 in the tray arm. The mean blood loss measured was 249 ml (SD: 170). The visual blood loss estimation was more accurate for the tray births compared to the deliveries where the drape was used (Figure 1).

Five (8%) women were identified as having more than 500 ml of blood loss, three in the drape arm and two in the tray arm. In two cases, additional intravenous oxytocin was given, and in the case where blood loss was >1000 ml, the parturient was transferred to the hospital after initial fluid resuscitation. The other three cases responded to routine intramuscular oxytocin, uterine massage, and suturing of vaginal tears. There was one other parturient who required transfer to the hospital for neonatal indications unrelated to maternal blood loss.

For each of the included births, there was one questionnaire completed by the birth attended and one by the birthing woman. Eight different birth attendants completed the questionnaires; one questionnaire was completed for each birth. The responses were documented on a tick sheet, and the questions were as described in Tables 3 and 4.

All the birth attendants found it easy to place the devices. For five parturients, the device placement was perceived as difficult (three of the drape births and two of the tray births). The parturients perceived both devices as comfortable.

Most birth attendants reported that the device was useful in monitoring ongoing blood loss. In the drape arm, the birth attendants found measuring blood loss very easy in 29 (97%) of the births, and in 1 (3%) birth, the attendant found it fairly easy. This is compared to 25 (76%) and 7 (21%) in the tray group. In one birth, the attendant found measuring blood loss “fairly difficult” using the tray.

Overall satisfaction among the birth attendants using the drape showed that 26 (87%) “would choose to use the device for future births” and four (13%) were “not so keen but would use it,” compared to 30 (91%) and three (9%), respectively, in the tray group. The parturients would be keen to use the device for future births in 28 (93%) of the drape births and 33 (100%) of the tray births.

The participating parturients (including those who experienced PPH) were a low-risk cohort with no identifiable antenatal risk factors for PPH.

## 4. Discussion

### 4.1. Main Findings

The aim was to assess and compare the utility and ease of use of the Brass-V drape and the MaternaWell tray for the collection of postpartum blood loss. Both devices were well-received by the birth attendants and parturients, with no statistical difference found between the two devices.

Ease of measurement favoured the Brass-V drape with its calibrations making direct measurement possible. The blood collected in the MaternaWell tray was poured into a measuring jug to confirm the volume. In clinical use, the calibrations on the tray itself are used for monitoring. Ease of placement favoured the MaternaWell tray. The Brass-V drape needs to be tied around the abdomen, making it cumbersome. Neither of these differences was statistically significant.

Typical of a low-risk cohort, there were no major complications due to blood loss in this study and only one participant was transferred to the hospital for further management. The blood loss was both estimated and measured by the birth attendants. Even with the devices to aid the visual impression, the estimation was still less than the measured blood loss. Visual estimation underestimates blood loss with worsening accuracy at larger volumes [9–11, 17]. There was a greater variation between estimated and final measured blood loss with the drape, as opposed to the tray where the blood collects directly into the calibrated chamber. With the Brass-V drape, blood pooling around the parturient means blood is not immediately measurable.

Heitkamp et al. studied severe acute maternal morbidity in the Metro East subdistrict of Cape Town (Khayelitsha, where this study was conducted, falls within the same subdistrict). They showed that 95/119 (75.6%) cases of major obstetric haemorrhage were referred from midwife obstetric units, such as site B. This underscores the importance of having an acceptable and useful device for monitoring blood loss and ultimately implementing early intervention [18].

The Brass-V drape was the device used for blood loss monitoring and early detection of PPH in the E-MOTIVE trial. There was, however, no mention of the perceived utility of the device from the birth attendants' and parturients' perspectives [8]. The 91.2% adherence to the treatment bundle in the E-MOTIVE trial [8], combined with the positive experiences shown in the index study, shows great promise for the routine implementation and use of blood loss monitoring devices.

### 4.2. Strengths of the Study

The random allocation allowed for comparisons between the two devices. The birth attendants and parturients were not biased by previous experience with either device. The study was conducted in a low-risk setting where the use of these devices would be most appropriate.

### 4.3. Limitations

The most significant limitation was that this was a single-centre study with a small sample size. The study was not powered to determine the difference between the two methods in detecting massive haemorrhage. We acknowledge that in the three cases where the blood loss was more than 500 ml, further intervention and management should have taken place according to the E-MOTIVE principles. The visual estimation of blood loss by birth attendants may have been influenced by the fact that the measured amount was evident when the visual estimations were made. The study did not explore the qualitative views and perspectives of the parturients and birth attendants in the use of the devices.

Although the effectiveness of the Brass-V drape has been proven and is recommended for use in LMIC, it is still not a common practice [8]. The cost of a Brass-V drape (a single-use device) is ZAR 110 (USD $6). Besides the repetitive costs, stocking and procurement in low-resource settings may prove a barrier to use in practice [14]. The MaternaWell tray (at the time of publishing) costs approximately ZAR 200 (USD $10) and can be used multiple times.

Recommendations for future research include a multicentre study of different levels of care and high-risk women. It will also be useful to explore a large sample size that is powered to allow for comparison of devices when there is a larger volume PPH. Exploring the qualitative views of birth attendants and parturients in using these devices will also add valuable insights.

The practical implications are that this study shows using a blood loss monitoring device routinely in low-risk settings is not only feasible but also acceptable and favoured by birth attendants and parturients. However, as this study was conducted in a specific MOU setting, the findings cannot be generalised to hospital settings or women with high-risk pregnancies; further studies should be conducted to explore the views of parturients and birth attendants in other settings.

In 2023, the WHO published recommendations on the assessment of PPH and the use of a treatment bundle for postpartum haemorrhage. Routine objective measurement of postpartum blood loss was recommended for all women giving birth [1]. Furthermore, one of the research recommendations this guideline made was the need to determine which method of objective measurement of blood loss was most acceptable to women and health workers. It is here that our study adds value in highlighting these perspectives.

The measurement of postpartum blood loss has been at the forefront of current strategies to combat postpartum haemorrhage. There has been no gold standard to quantify blood loss. Visual estimation is proven to be inaccurate and underestimates blood loss [11]. The E-MOTIVE study showed that using a calibrated drape to detect PPH combined with a bundle of treatment and supported by a multifaceted implementation strategy resulted in a lower risk of severe postpartum haemorrhage, postpartum laparotomy, or maternal death from bleeding [8].

## 5. Conclusion

Both the MaternaWell tray and the Brass-V drape were perceived as acceptable and useful to the birth attendants and parturients. Both devices will lead to prompt and accurate diagnosis of postpartum blood loss. The MaternaWell tray shows promise for LMIC as it is reusable, affordable, and environmentally friendly. Its use in women with specific risk factors, such as obesity, needs further investigation. The perceived utility and ease of use of the Brass-V drape and the MaternaWell tray justify further research to determine the accuracy of blood loss quantification and the impact of monitoring and treatment bundles in both high- and low-risk participants.

## Figures and Tables

**Table 1:** Demographic details of participants.

Characteristic	All *N* = 63	Brass-V drape *N* = 30	MaternaWell tray *N* = 33	*P* value
Age in years Med^1^ (IQR^2^)	28 (24–33)	29 (25–33)	27 (23–31)	0.37
Parity 0 (*N*, %)	22/63 (34.9)	8/30 (26.7)	14/33	0.19
Parity (Med, range)	1 (0–4)	1 (0–3)	1 (0–4)	0.46
Parity >3	0/63	0/30	0/33	—
Birth GA^3^ (Med, IQR)	39 (39–40)	39 (38–40)	39.57 (38.86–40.29)	0.43
Birth Hb^4^ g/dL (Med, IQR)	12.1 (11.1–13.1)	12.5 (11.1–13.1)	12.1 (11.2–13.1)	0.76
Booking Hb g/dL ≤ 11 (N, %)	19 (52.8)	11 (36.7)	8 (24.24)	0.28
BMI^5^ in kg/m^2^ (Med, IQR)	29.8 (24.9–34.3)	30.3 (25.6–33.7)	29.8 (24.9–34.3)	0.76
BMI ≥30 kg/m^2^ (N, %)	31 (49.2)	15 (50)	16 (48.5)	0.90

^1^Med = median; ^2^IQR = interquartile range; ^3^GA = gestational age; ^4^Hb = haemoglobin; ^5^BMI = body mass index.

**Table 2:** Birth outcomes of participants.

Characteristic	All *N* = 63	Brass-V drape *N* = 30	Maternawell tray *N* = 33	*P* value
Mean duration of the first stage of labour H : M^1^ (SD^2^)	9 : 38 (4 : 4)	9 : 09 (4 : 30)	10 : 04 (5 : 00)	0.46
Mean duration of the second stage (SD) of labour	0 : 47 (1 : 13)	0 : 36 (0 : 39)	0 : 57 (1 : 35)	0.27
Perineal tears all *N* (%)	20 (31.7)	14 (42.4)	6 (20)	0.14
Live born *N* (%)	63 (100)	30 (100)	33 (100)	
Birthweight gram (Med^3^, IQR^4^)	3200 (3000–3400)	3170 (2940–3220)	3200 (3000–3600)	0.07^∗^
5 minute Apgar (Med, IQR)	10 (10–10)	10 (10–10)	10 (10–10)	0.03
Transferred to the hospital	2	2	0	n/a

^1^H : M = hours : minutes. ^2^SD = standard deviation. ^3^Med = median. ^4^IQR = interquartile range. ^∗^Kruskal–Wallis.

**Table 3:** Responses to questionnaires by birth attendants.

Question	Brass-V drape *n* = 30	MaternaWell tray *n* = 33	*P* value
Would you choose to use the device for future births?			
(1) Yes, I would be keen to use it	26 (87%)	30 (91%)	0.70
(2) I will use it, but I am not that keen	4 (13%)	3 (9%)	
(3) I would prefer not to use it	0	0	
(4) I would not use it	0	0	
Was it easy or difficult to put the device in place?			
(1) Very easy	21 (70%)	24 (73%)	1.00
(2) Fairly easy	9 (30%)	9 (27%)	
(3) Fairly difficult	0	0	
(4) Not used	0	0	
Did the device help to monitor ongoing blood loss?			
(1) Helped a lot	29 (97%)	29 (88%)	0.42
(2) Helped a little	0	2 (6%)	
(3) Did not help	1 (3%)	2 (6%)	
(4) Did not use	0	0	
Was the device removed early?			
Yes	0	2 (6%)	0.49
No	30 (100%)	31 (94%)	
Was measuring blood easy?			
(1) Very easy	29 (97%)	25 (76%)	0.03
(2) Fairly easy	1 (3%)	7 (21%)	
(3) Fairly difficult	0	1 (3%)	
(4) Very difficult	0	0	
(5) Not used	0	0	
Were there any complications?			
Yes	1 (3%)	0	0.48
No	29 (97%)	33 (100%)	

**Table 4:** Responses to questionnaires by participants.

Question	Brass-V drape *n* = 30	MaternaWell tray *n* = 33	*P* value
Was it easy to place the device?			
(1) Very easy	22 (73%)	26 (79%)	0.75
(2) Fairly easy	5 (17%)	5 (15%)	
(3) Fairly difficult	3 (10%)	2 (6%)	
(4) Very difficult	0	0	
(5) Not used	0	0	
Did you feel comfortable with the device?			
(1) Very comfortable	30 (100%)	26 (79%)	0.01
(2) Fairly comfortable	0	7 (21%)	
(3) Very uncomfortable	0	0	
(4) Did not use	0	0	
Did you feel the device helped to keep you clean?			
(1) Helped a lot	27 (90%)	28 (85%)	1.00
(2) Helped a little	1 (3%)	2 (6%)	
(3) Did not help	2 (6%)	3 (9%)	
(4) Did not use	0	0	
I would choose to use the device for a future birth?			
(1) Yes, I would be keen to use it	28 (93%)	33 (100%)	0.22
(2) I will use it, but I am not that keen	1 (3%)	0	
(3) I would prefer not to use it	1 (3%)	0	
(4) I would not use it	0	0	

## Data Availability

The data used to support the findings of this study are available from the corresponding author upon request.

## Abbreviations

E-MOTIVE: Early detection, massage of the uterus, oxytocic drug, tranexamic acid, IV fluid examination, and escalation
LMIC: Low- and middle-income countries
MOU: Midwife obstetric unit
NCCEMD: National Committee on Confidential Enquiries into Maternal Deaths
OH: Obstetric haemorrhage
PPH: Postpartum haemorrhage
USD: United States dollar.

## Additional Points

*Definitions*. Acceptability: how well was the intervention received by the target population. Ease of use: how easily users can use the product. Overall satisfaction: how satisfied was the target population with the product. Low-risk pregnancy: pregnancies without any identified risk factor. Postpartum haemorrhage: blood loss from the genital tract of ≥500 ml within 24 hours after vaginal birth [1].

## Supplementary Materials

Supplementary Materials The South African Medical Research Council trail registration document and the Consort-2010 checklist for reporting a randomised trial are available in the supplementary material document.
