# Supplementary material for: Two Postpartum Blood Collection Devices: The Brass-V Drape and MaternaWell Tray—As Experienced by Birth Attendants and Birthing Women—A Questionnaire-Based Randomised Study
Source: Obstet Gynecol Int. 2024 Aug 14;2024:6605833. doi: 10.1155/2024/6605833 (PMC11338661; doi:10.1155/2024/6605833)
Supplement: Supplementary Materials — The South African Medical Research Council trail registration document and the Consort-2010 checklist for reporting a randomised trial are available in the supplementary material document. [file 6605833.f1.pdf]

CONSORT 2010 checklist of information to include when reporting a randomised trial*

Section/Topic	Item No	Checklist item	Reported on page No
**Title and abstract**			
	1a	Identification as a randomised trial in the title	X
	1b	Structured summary of trial design, methods, results, and conclusions (for specific guidance see CONSORT for abstracts)	X
**Introduction**			
Background and objectives	2a	Scientific background and explanation of rationale	X
	2b	Specific objectives or hypotheses	X
**Methods**			
Trial design	3a	Description of trial design (such as parallel, factorial) including allocation ratio	X
	3b	Important changes to methods after trial commencement (such as eligibility criteria), with reasons	N/A
Participants	4a	Eligibility criteria for participants	X
	4b	Settings and locations where the data were collected	X
Interventions	5	The interventions for each group with sufficient details to allow replication, including how and when they were actually administered	X
Outcomes	6a	Completely defined pre-specified primary and secondary outcome measures, including how and when they were assessed	X
	6b	Any changes to trial outcomes after the trial commenced, with reasons	N/A
Sample size	7a	How sample size was determined	X
	7b	When applicable, explanation of any interim analyses and stopping guidelines	N/A
Randomisation:			X
Sequence generation	8a	Method used to generate the random allocation sequence	
	8b	Type of randomisation; details of any restriction (such as blocking and block size)	X
Allocation concealment mechanism	9	Mechanism used to implement the random allocation sequence (such as sequentially numbered containers), describing any steps taken to conceal the sequence until interventions were assigned	X
Implementation	10	Who generated the random allocation sequence, who enrolled participants, and who assigned participants to interventions	X
Blinding	11a	If done, who was blinded after assignment to interventions (for example, participants, care providers, those	X

		assessing outcomes) and how	
	11b	If relevant, description of the similarity of interventions	N/A
Statistical methods	12a	Statistical methods used to compare groups for primary and secondary outcomes	X
	12b	Methods for additional analyses, such as subgroup analyses and adjusted analyses	N/A
**Results**			
Participant flow (a diagram is strongly recommended)	13a	For each group, the numbers of participants who were randomly assigned, received intended treatment, and were analysed for the primary outcome	X
	13b	For each group, losses and exclusions after randomisation, together with reasons	X
Recruitment	14a	Dates defining the periods of recruitment and follow-up	X
	14b	Why the trial ended or was stopped	N/A
Baseline data	15	A table showing baseline demographic and clinical characteristics for each group	X
Numbers analysed	16	For each group, number of participants (denominator) included in each analysis and whether the analysis was by original assigned groups	X
Outcomes and estimation	17a	For each primary and secondary outcome, results for each group, and the estimated effect size and its precision (such as 95% confidence interval)	X
	17b	For binary outcomes, presentation of both absolute and relative effect sizes is recommended	N/A
Ancillary analyses	18	Results of any other analyses performed, including subgroup analyses and adjusted analyses, distinguishing pre-specified from exploratory	X
Harms	19	All important harms or unintended effects in each group (for specific guidance see CONSORT for harms)	N/A
**Discussion**			
Limitations	20	Trial limitations, addressing sources of potential bias, imprecision, and, if relevant, multiplicity of analyses	X
Generalisability	21	Generalisability (external validity, applicability) of the trial findings	X
Interpretation	22	Interpretation consistent with results, balancing benefits and harms, and considering other relevant evidence	X
**Other information**			
Registration	23	Registration number and name of trial registry	X
Protocol	24	Where the full trial protocol can be accessed, if available	X
Funding	25	Sources of funding and other support (such as supply of drugs), role of funders	X

*We strongly recommend reading this statement in conjunction with the CONSORT 2010 Explanation and Elaboration for important clarifications on all the items. If relevant, we also recommend reading CONSORT extensions for cluster randomised trials, non-inferiority and equivalence trials, non-pharmacological treatments, herbal interventions, and pragmatic trials. Additional extensions are forthcoming: for those and for up to date references relevant to this checklist, see www.consort-statement.org.

South African National Clinical Trials Registry

South African Medical Research Council, Cochrane South Africa
PO Box 19070, Tygerberg, 7505, South Africa
Telephone: +27 21 938 0506 / +27 21 938 0834 Fax: +27 21 938 0836
Email: sactradmin@mrc.ac.za Website: sanctr.samrc.ac.za

**Trial no.:**	DOH-27-062022-9046	**Date of Approval:**	15/06/2022
**Trial Status:**	Approved		

TRIAL DESCRIPTION	
**Public title**	The utility and acceptability of a Brass V-drape versus a blood loss monitoring device for the collection of postpartum blood loss in low-risk term vaginal deliveries; a prospective parallel randomised trial.
**Official scientific title**	The utility and acceptability of a Brass V-drape versus a blood loss monitoring device for the collection of postpartum blood loss in low-risk term vaginal deliveries; a prospective parallel randomised trial.
**Brief summary describing the background and objectives of the trial**	A global effort is required to strengthen the management of postpartum haemorrhage (PPH) in low-income countries. There is no reliable clinical standard for the estimation of obstetric haemorrhage during delivery. The lack of an appropriate blood loss measurement may lead to delayed PPH recognition and management which may lead to significant morbidity and mortality. It is evident that more studies are needed in LMIC to quantify blood loss post vaginal delivery accurately and practically to prevent maternal morbidity and mortality. The study aims to assess the perceived usefulness and ease of use of a Brass V- drape versus blood loss monitoring device for the collection of postpartum blood loss. Robust data comparing the usefulness of different blood collection devices would be a major step to implement blood collection and measurement after birth.To assess and compare the perceived usefulness and ease of use of a Brass V-Drape versus the blood loss monitoring device for the collection of post-delivery blood loss by both A birth attendant as well as the participant (women giving birth).
**Type of trial**	RCT
**Acronym (If the trial has an acronym then please provide)**	
**Disease(s) or condition(s) being studied**	Obstetrics and Gynecology
**Sub-Disease(s) or condition(s) being studied**	Postpartum Haemorrhage
**Purpose of the trial**	Early detection /Screening
**Anticipated trial start date**	06/06/2022
**Actual trial start date**	
**Anticipated date of last follow up**	31/12/2022
**Actual Last follow-up date**	
**Anticipated target sample size (number of participants)**	60
**Actual target sample size (number of participants)**	
**Recruitment status**	Not yet recruiting
**Publication URL**	

**Secondary Ids**	**Issuing authority/Trial register**
23789	Stellenbosch University Health Research Ethics Committee 1

STUDY DESIGN

**Intervention assignment**	**Allocation to intervention**	**If randomised, describe how the allocation sequence was generated**	**Describe how the allocation sequence/code was concealed from the person allocating the participants to the intervention arms**	**Masking**	**If masking / blinding was used**
Parallel: different groups receive different interventions at	Randomised	Simple randomization using by using procedures such as coin-tossing or dice-rolling	Sealed opaque envelopes	Open-label(Masking Not Used)	

same time during study						

INTERVENTIONS						
**Intervention type**	**Intervention name**	**Dose**	**Duration**	**Intervention description**	**Group size**	**Nature of control**
Control Group	Brass V drape	not applicable	30 minutes	The birth attendant/midwife will place a waterproof draw sheet and the Brass V drape after the delivery of the baby. After delivery of the placenta and once bleeding has stopped or after 30 minutes has passed since placement, the blood collection device will be removed. The birth attendant will then estimate the spillage of blood on the draw sheet and measure the volume of blood by using scales and subtracting the known weight without blood loss. After completion of the delivery as well as blood collection both the midwife as well as the participant will be asked to complete a questionnaire, the midwife will oversee this.	30	Uncontrolled
Experimental Group	Blood monitoring device	not applicable	30 minutes	The birth attendant/midwife will place a waterproof draw sheet and the allocated device after the delivery of the baby. After delivery of the placenta and once bleeding has stopped or after 30 minutes has passed since placement, the blood monitoring device will be removed. The birth attendant will then estimate the spillage of blood on the draw sheet and measure the volume of blood using a standard measuring jug. After completion of the delivery as well as blood collection both the midwife as well as the participant will be asked to complete a questionnaire, the midwife will oversee this.	30	

ELIGIBILITY CRITERIA					
**List inclusion criteria**	**List exclusion criteria**	**Age Category**	**Minimum age**	**Maximum age**	**Gender**
Eighteen years and older Gestation of 37 weeks and more Singleton pregnancy Low risk pregnancies	Under the age of eighteen Gestation of 36+6 and less Multiple pregnancies Complicated pregnancies not meeting criteria for delivery at MOU but unintentionally deliver at the MOU including conditions such as hypertension, diabetes, previous caesarean sections	Adult: 19 Year(s)-105 Year(s)	18 Year(s)	45 Year(s)	Female

APPROVALS

Has the study received appropriate ethics committee approval	Date the study will be submitted for approval	Date of approval	Name of the ethics committee
Yes		06/01/2022	Health Research Ethics Comittee

Ethics Committee Address

Street address	City	Postal code	Country
Francie Van Zijl Drive, Tygerberg Medical Campus	Cape Town	7505	South Africa

Has the study received appropriate ethics committee approval	Date the study will be submitted for approval	Date of approval	Name of the ethics committee
Yes		06/01/2022	SAHPRA

Ethics Committee Address

Street address	City	Postal code	Country
CSIR Reception Building 38a Meiring Naudé Road Brummeria Pretoria	Pretoria	0083	South Africa

OUTCOMES		
**Type of outcome**	**Outcome**	**Timepoint(s) at which outcome measured**
Primary Outcome	To assess and compare the perceived usefulness and ease of use of a Brass V-Drape versus the blood loss monitoring device for the collection of post-delivery blood loss by both the birth attendant as well as the participant (women giving birth).	Once post-delivery blood loss collection has been completed
Secondary Outcome	To describe the characteristics of the study participants	After post-delivery blood loss collection is completed
Secondary Outcome	To assess the blood loss of each patient	After post-delivery blood loss collection is completed
Secondary Outcome	To review the details of patients with blood loss of > 500ml and identify potential risk factors for the blood loss.	After post-delivery blood loss collection is completed

RECRUITMENT CENTRES				
**Name of recruitment centre**	**Street address**	**City**	**Postal code**	**Country**
Khayelitsha Site B Midwives and Obstetric unit	Lwandle Road, Site B Khayelitsha	Cape Town	7784	South Africa

FUNDING SOURCES				
**Name of source**	**Street address**	**City**	**Postal code**	**Country**
South African Society of Obstetrics and Gynaecology	203 Beyers Naude Drive	Johannesburg	2118	South Africa

SPONSORS						
**Sponsor level**	**Name**	**Street address**	**City**	**Postal code**	**Country**	**Nature of sponsor**
Primary Sponsor	South African Society of Obstetrics and Gynaecology	203 Beyers Naude Drive	Johannesburg	2118	South Africa	Charities/Societies/Foundation

COLLABORATORS				
**Name**	**Street address**	**City**	**Postal code**	**Country**

CONTACT PEOPLE				
**Role**	**Name**	**Email**	**Phone**	**Street address**
**Principal Investigator**	Jade Monique Esau	jademe2020@gmail.com	+27763315545	30 Newfields Crescent, Newfields
**City**	**Postal code**	**Country**	**Position/Affiliation**	
Cape Town	7764	South Africa	Obstetric Registrar	
**Role**	**Name**	**Email**	**Phone**	**Street address**
**Public Enquiries**	Jade Monique Esau	jademe2020@gmail.com	+27763315545	30 Newfields Crescent, Newfields
**City**	**Postal code**	**Country**	**Position/Affiliation**	
Cape Town	7764	South Africa	Obstetric and Gynaecology Registrar	
**Role**	**Name**	**Email**	**Phone**	**Street address**
**Scientific Enquiries**	Liesl De Waard	ldewaard@sun.ac.za	+27738878366	Fransie van Zijl Avenue
**City**	**Postal code**	**Country**	**Position/Affiliation**	
Parow	7500	South Africa	Medical Specialist Obstetrics and Gynaecology	

REPORTING				
**Share IPD**	**Description**	**Additional Document Types**	**Sharing Time Frame**	**Key Access Criteria**
Yes	The investigators will be willing to share the individual participant data after de-identification of data (text,	Clinical Study Report,Informed Consent Form,Statistical Analysis Plan,Study Protocol	3 months after publication, 3 months to five years	Accredited researcher with methological sound proposals

	figures and tables etc) to researchers who provide methological sound proposals.			
**URL**	**Results Available**	**Results Summary**	**Result Posting Date**	**First Journal Publication Date**
	No			
**Result Upload 1:**	**Result Upload 2:**	**Result Upload 3:**	**Result Upload 4:**	**Result Upload 5:**

**Result URL Hyperlinks**	**Link To Protocol**
Result URL Hyperlinks	

Changes to trial information

**Section Name**	**Field Name**	**Date**	**Reason**	**Old Value**	**Updated Value**
SecondaryID	SecondaryID List	06/06/2022	was not added previously		23789 , Stellenbosch University Health Research Ethics Committee 1, 4787
**Section Name**	**Field Name**	**Date**	**Reason**	**Old Value**	**Updated Value**
Funding Source	FundingSources List	06/06/2022	previously unclear if needed to document as it was added under sponsors		South African Society of Obstetrics and Gynaecology, 203 Beyers Naude Drive, Johannesburg, 2118, South Africa, Charities/Societies/Foundation, , 19 657.06, Primary Funder
